# Custom-Fitted In- and Around-the-Ear Sensors for Unobtrusive and On-the-Go EEG Acquisitions: Development and Validation

**DOI:** 10.3390/s21092953

**Published:** 2021-04-23

**Authors:** Olivier Valentin, Guilhem Viallet, Aidin Delnavaz, Gabrielle Cretot-Richert, Mikaël Ducharme, Hami Monsarat-Chanon, Jérémie Voix

**Affiliations:** 1 École de Technologie Supérieure, 1100 Rue Notre-Dame Ouest, Montréal, QC H3C 1K3, Canada; gviallet@critias.ca (G.V.); adelnavaz@critias.ca (A.D.); gcretot@critias.ca (G.C.-R.); mducharme@critias.ca (M.D.); hchanon@critias.ca (H.M.-C.); jeremie.voix@etsmtl.ca (J.V.); 2 Centre for Interdisciplinary Research in Music, Media, and Technology, McGill University, 527 Rue Sherbrooke Ouest, Montréal, QC H3A 1E3, Canada

**Keywords:** silicone electrodes, wearables, brain computer interface (BCI), auditory steady-state response (ASSR), event-related potentials (ERP), electroencephalography (EEG)

## Abstract

Objectives: This paper aims to validate the performance and physical design of a wearable, unobtrusive ear-centered electroencephalography (EEG) device, dubbed “EARtrodes”, using early and late auditory evoked responses. Results would also offer a proof-of-concept for the device to be used as a concealed brain–computer interface (BCI). Design: The device is composed of a custom-fitted earpiece and an ergonomic behind-the-ear piece with embedded electrodes made of a soft and flexible combination of silicone rubber and carbon fibers. The location of the conductive silicone electrodes inside the ear canal and the optimal geometry of the behind-the-ear piece were obtained through morphological and geometrical analysis of the human ear canal and the region around-the-ear. An entirely conductive generic earpiece was also developed to assess the potential of a universal, more affordable solution. Results: Early latency results illustrate the conductive silicone electrodes’ capability to record quality EEG signals, comparable to those obtained with traditional gold-plated electrodes. Additionally, late latency results demonstrate EARtrodes’ capacity to reliably detect decision-making processes from the ear. Conclusions: EEG results validate the performance of EARtrodes as a circum-aural and intra-aural EEG recording system adapted for a wide range of applications in audiology, neuroscience, clinical research, and as an unobtrusive BCI.

## 1. Introduction

Electroencephalography (EEG) is a valuable tool for understanding brain function. It has been widely used for medical diagnoses, neurocognitive research and brain–computer interfaces (BCI) [1]. Conventional EEG systems measure the brain’s electrical activity using caps, which maintain electrodes in contact with the skull (scalp-EEG) and which are connected to wires transmitting the signals to differential amplifiers connected to a computer. State-of-the-art EEG recording requires trained clinicians, long preparation times of the skin surface to reduce skin–electrode electrical impedance, and a high-level laboratory infrastructure to provide controlled environments. In addition, wired EEG technology heavily restricts user mobility since any cable movements can greatly compromise signal quality. Moreover, EEG caps are uncomfortable to wear, impractical for daily-life situations and inadequate for social settings. These restrictions limit the research questions that can be addressed with conventional EEG and prevent its extensive potential for BCI applications [2].

The proposed ear-EEG acquisition system including intra-aural (inside the ear canal) and circum-aural (around the ear canal) EEG is a relatively new approach with user-friendly characteristics [3]. This approach can bring EEG into the consumer domain and make possible new BCI applications beyond the confines of the laboratory setting. Ear-EEG enables a less time-consuming setup process and could be the most discreet, least obtrusive, user-friendly and noninvasive solution to date. The ear canal is relatively hair free and exhibits geometrical asymmetry. These two characteristics significantly improve the electrical/mechanical contact between the skin and electrode and enhance the repeatability of the recordings. Moreover, the tight fit of a custom molded earpiece inside the ear canal applies pressure on the electrodes ensuring fixed electrode positions and a significant reduction in the motion artifacts that typically contaminate signal quality in conventional EEG.

Several intra-aural EEG platforms have been proposed in the last few decades. Silver electrodes embedded within a personalized earpiece placed in the outer ear was one of the first attempts at ear-EEG with results comparable to those obtained with conventional scalp-EEG for both auditory and visual evoked potentials and for steady-state and transient responses [4]. The proposed personalized ear-EEG concept was further investigated and validated over a larger population of subjects and for more EEG paradigms [5]. The same research team later introduced a generic earpiece based on a memory foam substrate and conductive cloth electrodes to address the issues of cost and ease-of-use associated with the personalized earpieces [6,7]. Shortly after, a dry contact ear-EEG electrode based on a titanium pin coated with iridium-oxide embedded within a soft individualized earpiece was prototyped and tested in four conventional EEG paradigms [8]. Around the same time, a lightweight in-ear biosensing system was introduced, able to continuously record the electrical activity of the human brain, eyes, and muscles concurrently using silver, fabric or copper electrodes placed on a generic earpiece [9]. Currently, a two-channel in-ear EEG system called Auris (CGX Systems, California, USA) with replaceable HydroFlex earbuds, is commercially available for sleep studies, exploratory research and long-term data collection.

In addition to these intra-aural EEG setups, several circum-aural EEG setups have been developed in recent years. A wireless behind-the-ear EEG recording device was proposed as an ambulatory EEG system [10]. The potential of ultra-thin and foldable electrodes laminated on the surfaces of the outer ear (the auricle) and adjacent regions (the mastoid) was explored for long-term, high-fidelity EEG recording of brain signals [11]. A reusable, flexible printed Ag/AgCl electrodes system consisting of ten electrodes arranged in a C-shape, known as the cEEGrid, was recently developed using flex-print technology to fit around the ear [12]. Many studies validated the performances of the cEEGrid to record reliable continuous EEG, event-related potentials and neural oscillations [12,13,14,15]. As well, automatic seizure detection with unobtrusive EEG electrodes placed behind the ear has been investigated and achieved [16].

A combination of intra-aural EEG and circum-aural EEG has also been the subject of a few studies. A system of miniaturized EEG electrodes integrated into a baseball cap (scalp-EEG) and an individualized silicone earpiece (intra-aural EEG) with additional electrodes placed behind and above the ears (circum-aural EEG) was proposed to obtain concealed EEG recordings with results comparable to conventional scalp EEG systems [17]. Similarly, a wearable in-the-ear EEG system including a gold-tip electrode inserted into the ear canal and two snap electrodes attached behind the ear was manufactured and tested with a steady-state visual-evoked potential paradigm [18].

However, none of the ear-EEG technologies reviewed thus far have optimized their prototypes in terms of the shape and electrode placement in order to maximize the quality of the skin–electrode contact and minimize the EEG signal artifacts due to head motion or jaw movement. Most of the ear-EEG technologies have been based on either integrating hard EEG electrodes into already manufactured customized earpieces or adding soft electrodes to available generic earpieces. In both cases, the resulting prototypes run the risk of an inconsistent assembly of electrodes and earpieces that could cause a deterioration of the quality of the skin–electrode contact and the comfort of the piece inside the ear canal. A few studies have tried to overcome this problem using a composite of carbon nanotubes with flexible material such as polydimethylsiloxane (CNT/PDMS) [19], graphene paper [20] or silicone rubber [21]. However, the manufacturing of these conductive parts involves elaborate chemical procedures, which could lead to high manufacturing costs, sacrifice flexibility for the sake of conductivity or suffer from the scarceness of details regarding relative quantities and type of materials to be mixed.

This study provides useful characteristics for ear-EEG, based on the 3D-scanning analysis of ear molds of two jaw end positions: opened and closed, which makes it possible to identify the optimized placement for the electrode within the earpiece. Moreover, the behind-the-ear piece was geometrically optimized and ergonomically designed to promote and maintain contact with the curved surfaces of the head around the ear without the need for a headband, adhesives or any of the other external equipment that is generally used with circum-aural EEG devices. In addition, the potential of conductive silicone, combining carbon fibers and medical-grade silicone rubber, is investigated as a promising material to make soft electrodes. A technique is proposed to standardize the manufacturing of conductive silicone electrodes and the molding process of the custom-fitted earpiece to produce an integrated and uniform soft electrode-earpiece structure dubbed EARtrodes: an intra-aural and circum-aural EEG device designed for BCI applications. Alternatively, a less customized and therefore more universal solution is also presented herein: fully conductive generic earpieces based on this conductive silicone electrode concept. For this study, these universal EEG earpieces were produced in three different sizes.

The perceived comfort of two intra-aural EEG earpiece designs is assessed using subjective bipolar comfort rating scales and the Auditory Steady-State Responses (ASSR) results are presented for the conductive silicone electrode earpieces. These results were compared to conventional gold-plated electrodes. Finally, Auditory Event-Related Potentials (AERPs) were recorded using circum- and intra-aural earpiece components to investigate the capacity of the EARtrodes to record ERPs such as the P300, a voluntary cognitive response, useful in the context of BCI applications.

## 2. Materials

### 2.1. Prototyping of Conductive Silicone Electrodes

The main design challenge for EARtrodes is the significant variability in shapes and sizes of ears among individuals. This has an impact on the mechanical design and the areas that electrodes have to reach: inside the ear canal, around the ear canal, including the auricle, mastoid and temporal bone. These variations need to be accommodated by a material that can easily be deformed or adapted to various angles, and that can be processed in different shapes and sizes. Silicone rubber has been selected as a base material because this material, as well as plastic and foam, is commonly used in the hearing protection industry. Several types of fillers or loading material have been investigated to make the silicone rubber conductive. The most important selection criterion for the filler is its simplicity of use and chemical procedures. Black carbon is chosen as filler for the EARtrodes application because of its affordability and high availability. Strands of chopped carbon fiber are first separated in a 70% isopropyl alcohol solution and then added to the A-component of a two-part medical grade silicone before mixing with the B-component. Then the mix of carbon-silicone is cured in a mold to obtain the desired shape and size. A range of 1.5% to 3% of carbon in the material (percentage of weight in the uncured silicone) is found to be sufficient for it to exhibit enough conductivity for EEG recording without denaturing the flexibility of the silicone rubber. The shore-A hardness value of the medical grade silicone was around 22 HRA before mixing and around 25 HRA after mixing. As a small portion of the mixed solution is used to make each electrode, it would be difficult to estimate the exact amount of carbon that each electrode contains at the end of the manufacturing process. The distribution of the carbon in the solution may vary, varying the amount of carbon in the electrodes, but it should remain as uniform as possible.

### 2.2. Intra-Aural Earpiece

The ear canal is a dynamic environment that is momentarily deformed by the movement of the temporomandibular joint (TMJ), such as, when eating, chewing, speaking, etc. These movements may break the electrode-skin contact and reduce the quality of EEG signals. The amount of mechanical deformation is not the same throughout the ear canal and may vary from one participant to another, as reported in [22]. The study investigated the ear canal’s dynamic deformations for five participants using custom-fitted ear molds in open and closed-jaw positions. The custom-molded earplugs were digitally scanned using a laser Coordinate Measuring Machine (CMM, Mitutoyo, Japan). The 3D images of ear molds for each participant were later aligned by PolyWorks (InnovMetric Software Inc., Québec, QC, Canada) to find the best fit between open-jaw and closed-jaw ear molds. The results show that for all participants, at least two distinct regions are potentially suitable for the placement of EEG electrodes. The electrode positions were chosen based on the following criteria:

- The electrodes are close neither to the tip of the earplug nor to the concha of the ear since they would likely not be in contact with the ear canal at these two regions;
- The electrodes are preferably placed in nearly flat or large curvature regions of the ear canal;
- The choice of the relative positions of two electrodes in one ear mold should ensure the maximum possible distance between them;
- The electrodes are placed in an area with zero or most preferably positive deformations, so that, regardless of the jaw’s position, the electrode always stays in contact with the skin of the ear canal.

**Figure 1:**
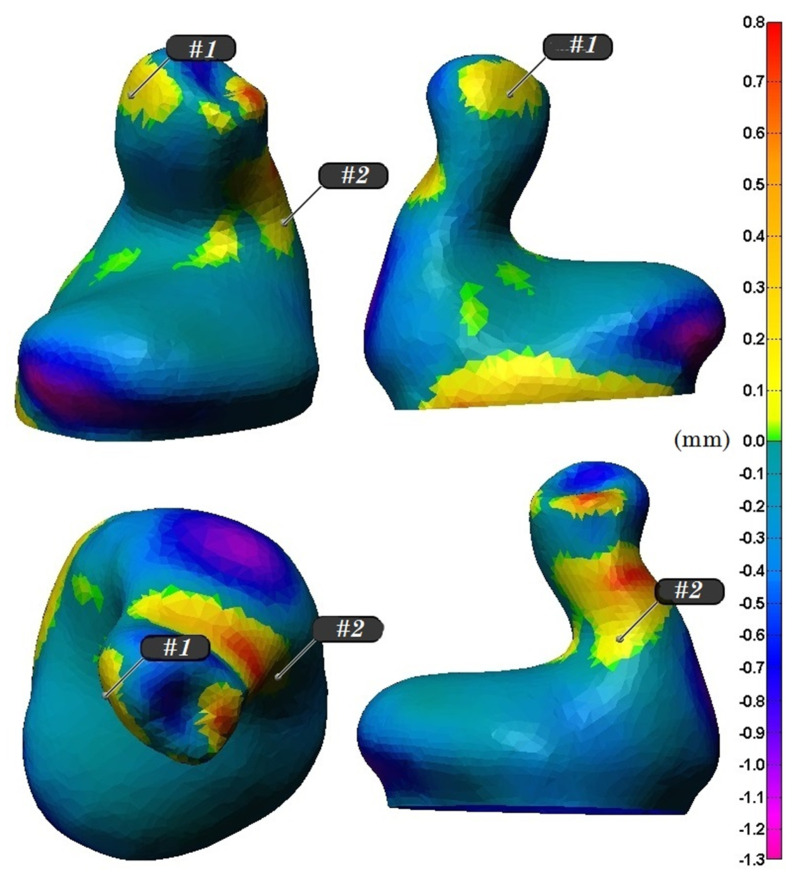
Two optimal electrode positions determined for one participant. The colored zone corresponds to the deformation field of the open-jaw custom fitted earpiece in relation to the closed-jaw earpiece.

The proposed electrode positions for one participant are illustrated in Figure 1.

**Figure 2:**
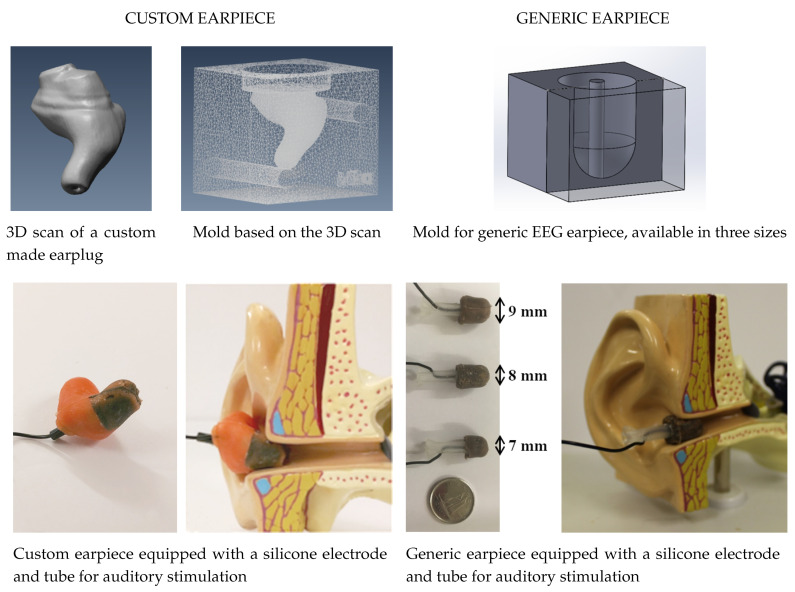
Conductive earpieces for intra-aural EEG recording. **Left**: custom-molded earpiece with conductive carbon-loaded silicone inserts; **Right**: fully conductive generic earpiece made of carbon-loaded silicon.

Based on these guidelines for electrode positioning, a rigid two-part mold corresponding to the negative shape of the custom-molded earpiece in the closed-jaw position is manufactured. The obtained mold is first filled with conductive silicone to obtain a fully conductive earpiece, which is subsequently shaped to perfectly match the predetermined positions of the electrodes. The wired pieces of electrodes are returned to their positions in the mold and a 1-mm diameter tube is placed in the centerline of the mold to let the auditory stimulus pass through the earpiece once molded. The final step of the molding assembly of the electrode-earpiece sensor is an overmolding process during which regular silicone rubber is injected resulting in a uniform piece. As for the fully conductive generic electrode-earpiece sensors, they are molded in a cylindrical shape overhung by a dome at the tip. Three different sizes are available, as shown in Figure 2. The participant can try and choose the size that comfortably fits his or her ear canal.

### 2.3. Circum-Aural Earpiece

**Figure 3:**
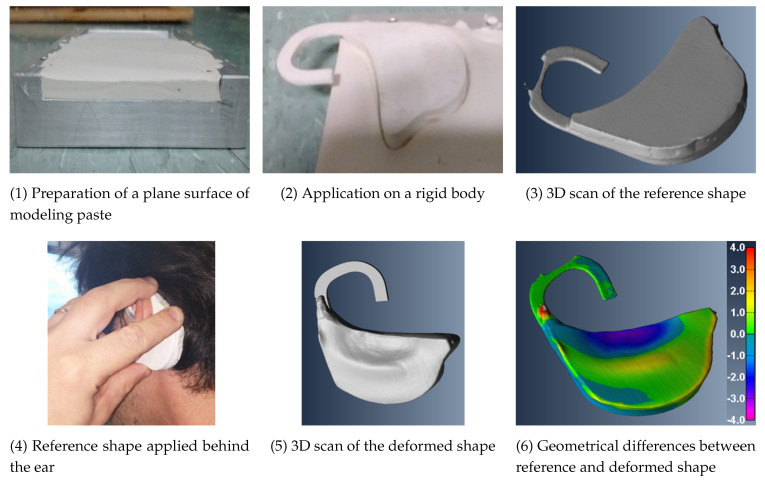
Practical steps to obtain the custom shape factor of the behind-the-ear piece.

To record circum-aural EEG signals with the conductive silicone electrodes, a behind-the-ear piece is required to hold the electrodes in place and keep them in contact with the skin. The most important criterion here is that the device ensure steady contact between the skin and the electrodes for the entire duration of the EEG recordings. An anthropomorphic study was performed to characterize the shape of the device. To obtain the precise shape of the behind-the-ear area, the curved surface of the head around the ear was molded using a professional modeling paste as shown in Figure 3. Step 1: A plane surface of the modeling paste is placed on a table; step 2: a molding blank is prepared using a neutral rigid 3D-printed sample; step 3: the 3D scan of the blank to be used for later reference was obtained using an EinScan-S 3D scanner (Shinning3D, Hangzhou, Zhejiang, China); step 4: The molding blank was placed in its position around the ear and pressed onto the surface of the head using mild pressure to imprint the behind-the-ear area of the participant; step 5: The resulting impression was 3D scanned; and step 6: it was compared to the geometric reference plane of the modeling paste. The geometrical differences between the blank reference and the behind-the-ear impression describe the precise shape of the behind-the-ear area.

**Figure 4:**
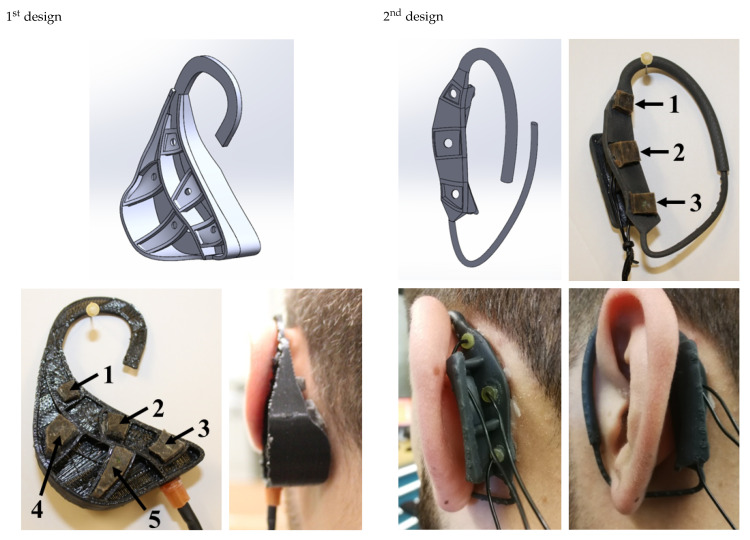
Initial (1st design on the **left**) and final (2nd design on the **right**) design of the behind-the-ear piece with embedded conductive silicone electrodes.

The behind-the-ear impression process was completed for eight individuals (six males, two females), aged between 21 and 37, with a height between 1.65 m and 1.87 m, all Caucasian. It yielded sixteen (right and left ear) different 3D models of the behind-the-ear morphology. The geometrical models were compared using PolyWorks (v11.0.34, module IMinspect). Morphing geometric tools combined with the similar geometric patterns observed on the sixteen models were used to obtain a unique average-geometry behind-the-ear model. Small cavities were then added to the model to support the conductive silicone electrodes. Two different versions of the behind-the-ear piece are illustrated in Figure 4. The first version is suitable for EEG recordings; however it requires the use of a headband to press the electrodes against the skin and to reach acceptable electrical impedance for the skin–electrode contact. Given the variety of behind-the-ear geometries among the participants, it was difficult to obtain high quality signals for all embedded electrodes.

The first version was subsequently modified by reducing it to the zones associated with electrode number 1, 2 and 3 as shown in Figure 4. The general shape of the second version is still based on the average geometry of the behind-the-ear area. It also contains a V-shape folded structure as shown in Figure 4 to support the behind-the-ear piece against the back of the pinna using a spring effect that efficiently presses the electrodes against the skin. In addition, a hook was added to the design to loop around the pinna and steady the behind-the-ear piece in its position. The frame was 3D printed with a stereo-lithography printer (Form2, Formlabs, Somerville. USA) in a flexible acrylic resin.

The first version was used for pretests and a proof-of-concept and the second version was used to obtain the results presented in this paper. The second design substantially reduced the ergonomic problems associated with the poor skin-contact of some electrodes for most of the participants.

### 2.4. Custom EEG Research Platform

**Figure 5:**
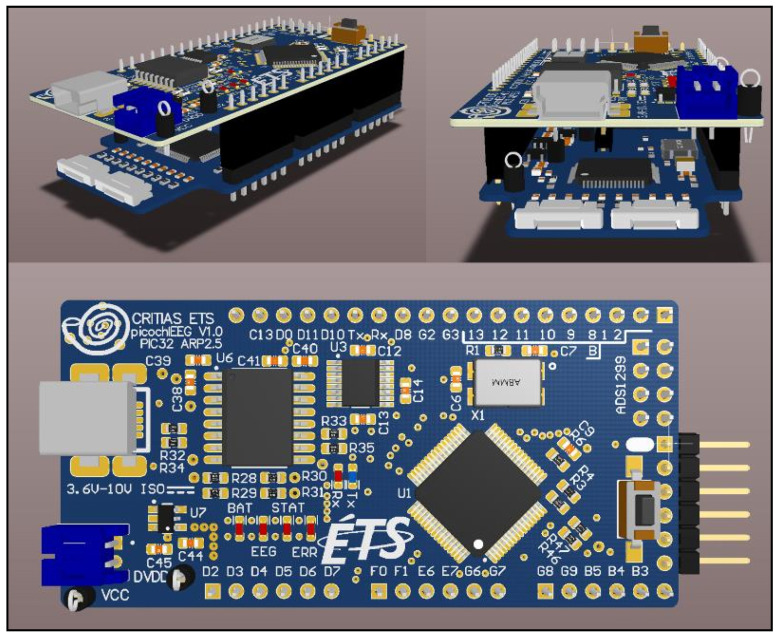
3D rendering of CochlEEG portable EEG amplifier.

The early and late auditory responses presented in this paper were recorded using a custom portable EEG platform, shown in Figure 5, designed by the authors. This mobile EEG research platform, dubbed CochlEEG, uses Texas Instruments’ ADS1299 (Dallas, TX, USA) specialized chip. It features eight acquisition channels (besides the two channels reserved for reference and ground electrodes) with variable sampling frequencies from 250 Hz to 4 kHz. Each acquisition channel has user-configurable low-noise 24-bits simultaneous-sampling delta-sigma analog-to-digital converter with a built-in programmable gain amplifier. CochlEEG uses a configurable DC-bias circuit, commonly called *Driven Right Leg* circuit, for improved common-mode rejection ratio (CMRR). CochlEEG supports wet and dry electrodes and has a bipolar 2.5 V output supply available for active electrodes. Data transmission is handled through a 5 kV isolated USB to serial integrated chip. Its software is used for channel and device configuration and converts the incoming data into a Lab Streaming Layer (LSL) data stream [23]. An in-depth technical overview of this custom EEG research platform is reported in [24].

## 3. Methods

Four experimental studies involving human participants were conducted to validate a proof-of-concept for the use of the device as an unobtrusive BCI:

- The first study aimed to assess the comfort of a custom in-ear piece versus a generic in-ear piece with a less complex design.
- The second study aimed to validate the conductive silicone material by comparing the EEG data recorded with conventional gold-plated electrodes to those obtained with conductive silicone electrodes.
- The third study aimed to demonstrate the capability of CochlEEG in recording EEG data at 4 kHz, in addition to the 0.5 kHz, 1 kHz and 2 kHz sampling frequencies whose validation was presented in previous work [24].
- Finally, the fourth study aimed to demonstrate the device’s capability to reliably detect decision-making processes through an event-related potential (ERP) generated by an auditory oddball task with the behind-the-ear piece used concurrently with an in-ear piece.

These studies were reviewed and approved by the *Comité d’éthique pour la recherche*, the Internal Review Board at *École de technologie supérieure (ÉTS)* in Montréal, Canada. Informed consent was obtained from all participants before they were enrolled in the study.

### 3.1. Study #1: Comfort Evaluation

**Figure 6:**
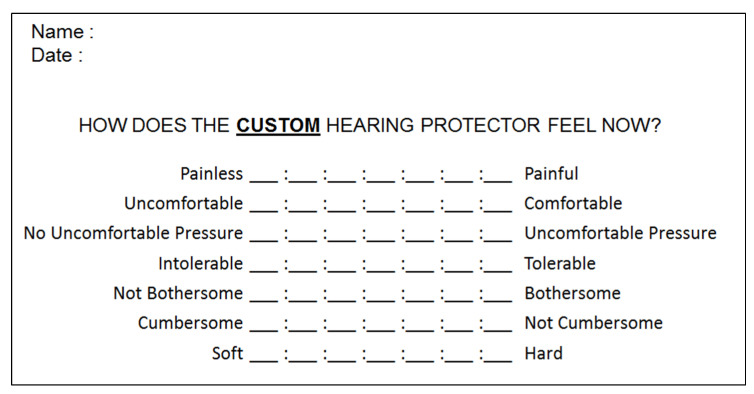
Customized version of the bipolar comfort rating scales, initially developed by Park and Casali for hearing protection devices. The same form was used to subjectively assess the comfort of the custom molded earpiece and of the generic in-ear piece.

The main goal of this brief comfort study was to determine if there is a difference between the custom in-ear piece and the generic in-ear piece, in terms of comfort. Ten individuals (six males, four females) aged between 22 and 34 participated in this first study. Half of the participants were randomly assigned to first wear the custom in-ear piece and then the generic in-ear piece. The other half wore the generic in-ear piece first and then the custom in-ear piece. After 15 min of wearing time, the perceived comfort of each in-ear piece was subjectively assessed using a customized version of the bipolar comfort rating scales (Figure 6) developed by Park and Casali for hearing protection devices [25].

Participants’ responses to the rating scales were converted to a numerical value ranging from 1 (closest to the left-end adjective) to 7 (closest to the right-end adjective) for lines 2, 4 and 6 and from 1 (closest to the right-end adjective) to 7 (closest to the left-end adjective) for lines 1, 3, 5 and 7. The individual comfort indexes were computed for each participant and each in-ear piece as the linear sum of the seven scales, ranging from 7 (most uncomfortable) to 49 (most comfortable). For comparative purposes, let us note that in their study on the comfort of hearing protectors, Park and Casali reported a mean comfort index of respectively 28 and 29 for the 3M CLASSIC and the 3M ULTRAFIT, two types of earplugs widely used in industrial workplaces.

### 3.2. Study #2: Validation of the Conductive Silicone

The aim of the second study is to validate the conductive silicone material. To do so, Auditory Steady-State Responses (ASSRs) obtained with conventional gold-plated electrodes were compared to those obtained with conductive silicone electrodes.

#### 3.2.1. ASSRs

ASSRs are electrophysiological responses evoked by modulated tones, which can be used for objective hearing threshold estimation of young children, older patients, and patients with cognitive deficits or behavioral disorders [26,27]. ASSR stimuli consist of two multiplied sine waves, the one with the highest frequency being the carrier frequency ($f_{c}$) corresponding to the audiometric frequency to be tested, while the one with the lowest frequency corresponds to the modulating envelope ($f_{m}$). In practice, amplitude variations induced by the modulation frequency stimulate the specific area in the cochlea corresponding to the carrier frequency. This phase-locked brain activity to the modulation frequency can be easily extracted from the EEG spectrum; a peak proportional to the stimulation level will manifest at the modulation frequency, and may also appear at its harmonics [28].

Historically, ASSRs were first elicited using modulation frequencies in the 40 Hz range [29]. A large number of studies reported that ASSRs recorded using modulation frequencies greater than 70 Hz are significantly less sensitive to arousal effects than those collected using modulation frequencies around 40 Hz. However, in the case of alert/awake adults, an ASSR evoked using stimuli modulated at 40 Hz present higher amplitudes and better signal-to-noise ratios, which might provide faster and more efficient ASSR detection [30,31].

#### 3.2.2. Participants

Ten individuals (six men and four women aged between 22 and 34) participated in the second study. They were recruited among students and colleagues. All participants were normal-hearing (no hearing threshold below 25 dB HL between 125 and 8000 Hz using tonal audiometry) and were free of past or present neurological conditions.

#### 3.2.3. Auditory Stimuli

In the second experiment, the stimuli were sinusoidal tones with a carrier frequency of 1000 Hz ($f_{c}$) that were 100% amplitude-modulated at 41 Hz ($f_{m}$). Equation (1) details the full mathematical formula used for stimuli generation:

(1) $$y\left(t\right)=\frac{\sin(2\pi f_{c}\left(t\right))}{2}\times \frac{\sin(2\pi f_{m}\left(t\right))+1}{2}$$

All stimuli were generated using a MATLAB R2015b program (Mathworks, Natick, MA, USA) script. They were amplified using an AC40 audiometer (Interacoustic, Middelfart, Danemark) before being binaurally presented via E-A-RTONE model 3A insert earphones (Aearo Technologies LLC, Indianapolis, IN, USA) at a stimulation level of 75 dB SPL. Stimuli calibration was performed using a B&K2128 Head and Torso Simulator (Brüel & Kjær, Nærum, Danemark).

#### 3.2.4. Recordings

**Figure 7:**
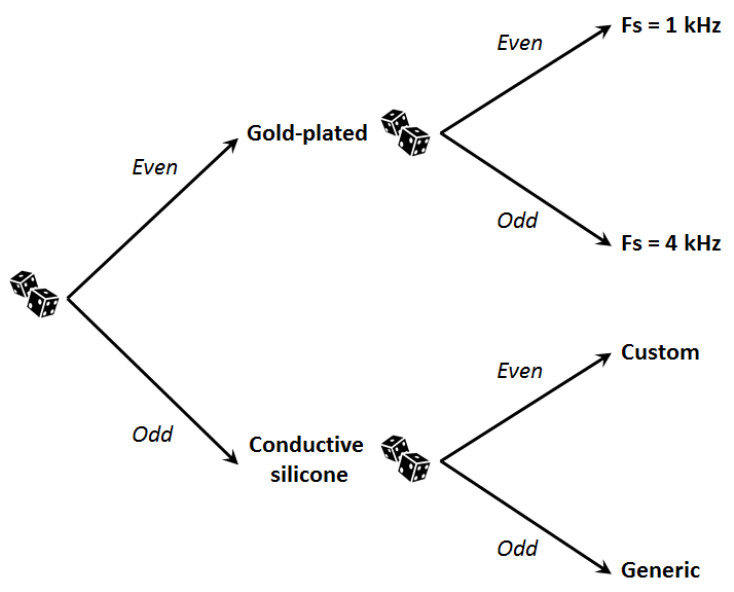
Tree diagram used to randomly determine the recordings’ order for each individual participant.

During all the measurements, participants remained seated on a comfortable ergonomic chair inside a double-walled audiometric booth. ASSR data were collected either using traditional gold wet electrodes or wet electrodes made of conductive silicone. The recording order was randomly determined for each individual participant using a six-sided dice and a tree diagram (Figure 7).

Traditionally, ASSR data are collected from an electrode placed at the vertex (Cz) using an electrode on the back of the neck (below the hairline) as reference and an electrode on the clavicle as ground. Since the aim of this second study is to explore the capability of using conductive silicone as in-ear electrodes, the exploring, ground and reference electrodes were placed at the vertex (Cz), on the lobe and into the ear canal respectively.

For the traditional gold electrodes, the exploring electrode consists of a gold-plated cup electrode and the ground electrode is composed of two gold-plated cup electrodes mounted like an ear clip, and the reference electrode is a gold foil electrode with earphone foam tips. For the conductive silicone electrodes, the exploring electrode is in the form of a disc snapped to a lead wire, the ground electrode consists of a silicone pad mounted like an ear clip, and the reference electrode is either a custom earplug with a conductive silicone electrode or a fully conductive generic earplug. The recording sites were cleaned using Nuprep abrasive gel (Weaver and Company, Aurora, CO, USA) and alcohol prior to the recording. The conductivity between the skin and all electrodes was ensured using Ten20 conductive paste (Weaver and Company). The interelectrode impedance were below 20 kΩ at 10 Hz for the traditional gold electrodes and below 60 kΩ at 10 Hz for the conductive silicone electrodes.

For the second study, ASSR data were recorded with a sampling rate of 1000 Hz using the custom EEG research platform presented in Section 2.4. Synchronization between stimulation and recording was performed using Lab Streaming Layer (LSL). Data analysis was performed offline using MATLAB software and the toolbox EEGLAB [32]. A 0.1–500 Hz FIR band-pass filter was applied to ASSR data before extraction of the data sweep. A total of six sweeps of sixteen epochs each with 1000 points per epoch were extracted from the data stream, averaged in the time domain, and then translated in the frequency domain using FFT.

### 3.3. Study #3: Validation of CochlEEG’s 4 kHz Sampling Rate

The aim of the third study is to validate the capability of CochlEEG to record EEG data at 4 kHz, in addition to the 0.5 kHz, 1 kHz and 2 kHz sampling frequencies whose validation was presented in previous work [24]. To do so, ASSR data obtained using conventional gold-plated electrodes with a sampling rate of 1000 Hz were compared to those obtained with a sampling rate of 4000 Hz. Ten individuals (six men and four women aged between 22 and 34) participated in the third study. They were recruited among students and colleagues. All participants were normal-hearing (no hearing threshold below 25 dB HL between 125 and 8000 Hz using tonal audiometry) and were free of past or present neurological conditions.

In the third experiment, the stimuli were sinusoidal tones with a carrier frequency of 1000 and 4000 Hz that were 100% amplitude-modulated at 41 Hz. The methods used to calibrate, generate, and present the stimuli were the same as the ones described in Section 3.2.3.

Since the aim of this third study is to validate the capability of CochlEEG in recording EEG data at 4 kHz, ASSR data were collected using traditional gold electrodes with a sampling rate of either 1000 Hz or 4000 Hz (the order of presentation being random). The exploring, ground and reference electrodes were placed at the vertex (Cz), on the ear lobe and in the ear canal respectively. The exploring electrode consists of a gold-plated cup electrode, the ground electrode is composed of two gold-plated cup electrodes mounted like an ear clip and the reference electrode is a gold foil electrode with earphone foam tips. All interelectrode impedance were below 20 kΩ at 10 Hz.

The preparation method, recording method, and filtering parameters were the same as the ones described in Section 3.2.4.

### 3.4. Study #4: Event-Related Potentials (ERPs)

#### 3.4.1. ERPs and the Oddball Paradigm

Event-related potentials (ERP) are important in EEG research because they are caused by the presentation of a stimuli. They translate the brain activity’s response to a specific event and are time-locked to that event, making them easier to isolate and study. Depending on the stimulation, you can expect these predictably timed peaks and troughs in voltage amplitude, which correspond to well-known and well-established cognitive processes [33]. Many ERPs are described by a letter and a number to indicate the type of polarization they represent (Negative or Positive) and the latency or order in which they are found. In this study, we are interested in the P3 or P300 response, a large positive amplitude wave occurring around 300 ms after a task-relevant stimuli is presented. The P300 response indicates that the subject recognizes the stimuli as a target, meaning it is related to decision-making. This makes ERPs and in particular the P300 most relevant for brain–computer interfaces (BCI) because it is a conscious and voluntary cognitive response [34]. It was therefore important to use a P300-generating paradigm in this study to demonstrate the capability of the new electrodes to reliably detect ERPs, validating their compatibility for the proposed ear-centric EEG acquisition platform.

For this study, an auditory oddball paradigm was chosen to measure a P300 response. Participants heard a series of repetitive tones, interrupted shortly and randomly by a deviant tone clearly higher in frequency. They were asked to pay attention to the deviant tones as targets and to count their occurrence while ignoring the standard stimuli [35]. The details of this task are derived from the work of Bennington et al. [36].

#### 3.4.2. Participants

Nine individuals (six males, three females) with ages ranging from 21 to 27, and hearing thresholds below 25 dB HL, were recruited to participate in the fourth study. All participants were free of past or present neurological conditions.

#### 3.4.3. Auditory Stimuli

ERPs were elicited using 40 auditory sequences. Each sequence consists of 10 sinusoidal tones of 69.8 ms duration (9.9 ms rise/fall and 50 ms plateau) of either 1000 Hz (standard) or 2000 Hz (target). Each stimulus was separated by a 2 s interstimulus interval and each sequence was separated by a 4-s intersequence interval. All stimuli were standard stimuli except for one that was a target stimulus randomly chosen at either the 7th, 8th, 9th or 10th position. The stimulation level was adjusted to a participant-controlled comfortable loudness, and E-A-RTONE model 3A insert earphones (Aearo Technologies LLC, Indianapolis, IN, USA) were used to deliver the stimuli.

#### 3.4.4. Recordings

EEG data were collected using five conductive silicone wet electrodes (three exploring electrodes + two electrodes for the ground and reference). The three electrodes of the behind-the-ear piece were placed around the left ear as exploring electrodes. The ground electrode was positioned on the right earlobe and the reference electrode was placed on the earpiece inserted in the left ear canal. The recording sites were cleaned with alcohol prior to the recordings and the conductivity between skin and electrodes was ensured using a conductive paste (Ten20, Weaver and Company). Electrode impedance was maintained below 60 kΩ at 10 Hz. For three participants, the impedance of some exploring electrodes could not be lowered below 60 kΩ; in these cases, the exploring electrodes were disabled. Table 1 details the number of exploring electrodes used for each participant. No online rejection threshold was applied and data were not filtered online, aside from the built-in anti-aliasing filter embedded in the hardware.

During ERP recordings, participants were seated in a dimly-lit double-walled audiometric booth. They were asked to respond to the target tones by pressing a button, to silently count the number of target occurrences, and to report the total number at the end of the recording session.

To analyze the oddball data, continuous time series were high-pass filtered at 0.1 Hz (zero phase filter order 500), resampled at 128 Hz, and low-pass filtered at 30 Hz (filter order 100). They were then epoched from −500 to 1000 ms and baseline corrected (−500 to 0 ms). Data recorded with less than two acquisition channels were not included in the analysis (i.e., participant S6). Artifact removal was performed using the EEGLAB 2019 toolbox as follows: An independent component analysis (ICA) was performed [37] on the data of participants who had all three exploring electrodes active at all times, generating three components. For these participants, one main eye-blink artifact component was identified for rejection using the component data scrolls and looking for obvious eye blinks and longitudinal eye movements. The projections of this component were then removed from each of the three electrode time series data. Additionally, for every participant, improbable muscular artifacts were identified using the probability and kurtosis criteria (standard deviation: 3) and rejected from further analysis (3%-to-10% of epochs were rejected using this criterion). After this preprocessing phase, the time series data was passed through a moving average FIR filter (zero phase filter order 8) to smooth out the data.

## 4. Results

### 4.1. Comfort of the In-Ear Pieces

**Figure 8:**
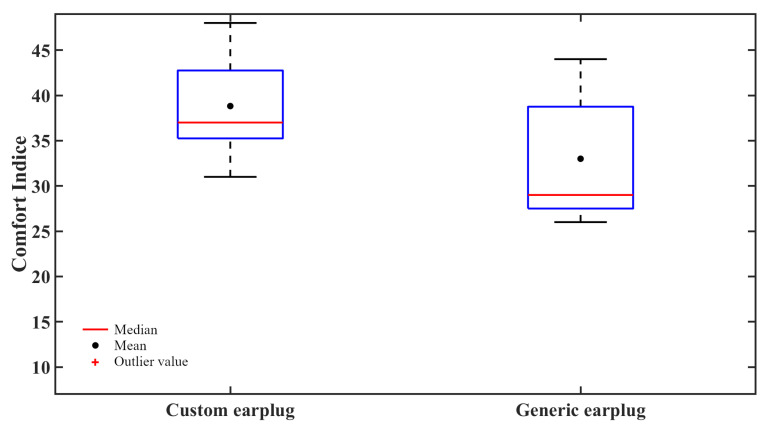
Comfort indices for the custom instrumented earplug and generic instrumented earplug used in this study show similar comfort rankings.

According to the comfort indices presented in Figure 8, the custom in-ear piece seems to be more comfortable than the generic in-ear pieces. This observation is supported by a two-sample *t*-test for noninferiority [38] computed using R 3.6.1 with MASS 7.3–51.4 [39], which rejects the null hypothesis at a 5% significance level (*p* = 1.84 × $10^{-6}$ with a margin tolerance ε=±σ) and confirms the idea that the comfort of the custom in-ear device is noninferior to the comfort of the generic in-ear device, whereas a two-sample *t*-test for nonsuperiority fails to reject the null hypothesis at a 5% significance level (*p* = 0.1113 with the same margin tolerance ε=±σ), supporting the idea that the comfort of the custom in-ear device is superior to the comfort of the generic in-ear device. However, a more extensive study, using a greater number of participants, should be conducted to validate this observation.

### 4.2. Validation of the Conductive Silicone Material

**Figure 9:**
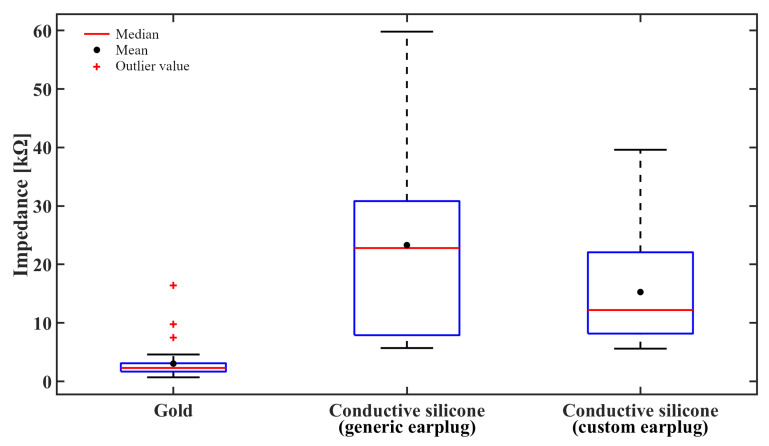
Interelectrode impedances measured for each electrode configuration. Impedances are much higher when using conductive silicone (C.S.) electrodes.

The interelectrode impedances presented in Figure 9 suggest that the impedances are much higher when using conductive silicone electrodes instead of gold-plated electrodes. A Friedman rank sum test [40] procedure confirms that gold-plated electrodes and conductive silicone electrodes are not equivalent with respect to impedances as this test rejects the null hypothesis at a 5% significance level (*p* = 3.773 × $10^{-12}$).

**Figure 10:**
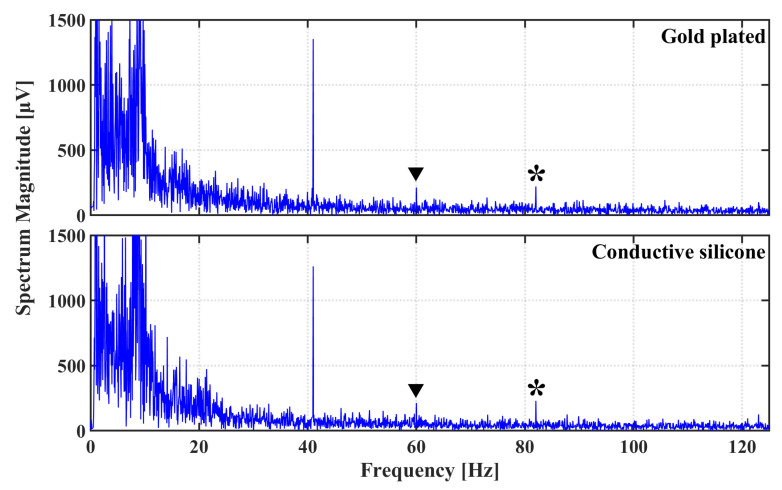
Power spectrum of EEG signals recorded on subject #3 with gold-plated electrodes (**top**), and conductive silicone electrodes (**bottom**). Peaks indicated with asterisks correspond to the second harmonics of the 41 Hz responses, which are particularly visible in this subject. Power line artifacts are indicated by solid triangles.

**Figure 11:**
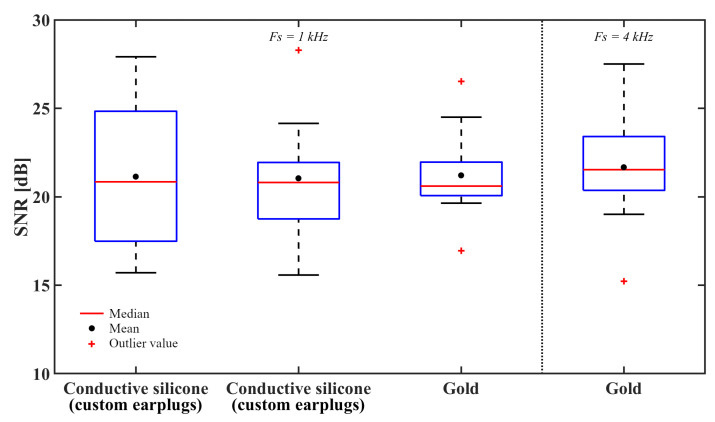
Average ASSR signal-to-noise ratios, in dB, collected from ten participants.

However, even if the impedances remain higher when using conductive silicone electrodes, results presented in Figure 10 and Figure 11 seem to indicate that the ASSR power spectrum and signal-to-noise ratios (SNRs) obtained using conductive silicone electrodes seem comparable to those obtained using gold-plated electrodes. This observation is confirmed by a Friedman rank sum test, which fails to reject the null hypothesis at a 5% significance level (*p* = 0.9048), providing strong evidence that ASSRs collected with conductive silicone electrodes are not statistically different to those collected using gold-plated electrodes.

### 4.3. Recordings at 4 kHz

CochlEEG was previously presented in a paper by the authors as a portable EEG data acquisition platform able to reliably record good quality EEG data with sampling rates of up to 2 kHz [24]. Thanks to a recent update of the firmware, the 4 kHz sampling frequency is now fully functional. Therefore, it was of interest to the authors to demonstrate the CochlEEG’s recording capability of EEG data at 4 kHz. Results in Figure 11 indicate that SNRs obtained with CochlEEG at 1000 Hz seem comparable to those obtained at 4000 Hz. This observation was confirmed by a Friedman rank sum test, which fails to reject the null hypothesis at a 5% significance level (*p* = 0.999), providing strong evidence that ASSRs collected at 1000 Hz are not statistically different from those collected at 4000 Hz.

### 4.4. Event-Related Potentials (ERP)

**Figure 12:**
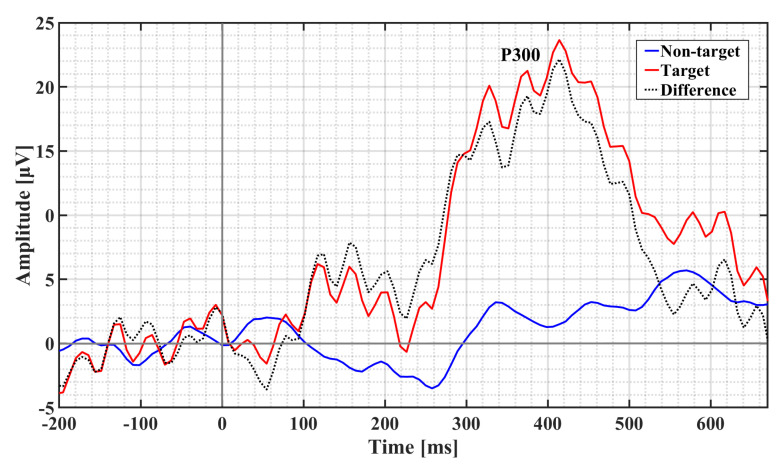
Grand average ERP (*N* = 8) observed in and around the ears.

The target detection error rates were minimal (mean of 1.875%) and demonstrated that participants were able to easily distinguish the target stimuli from the standard stimuli during the auditory oddball experiment. The grand average ERP is reported in Figure 12. The difference wave shows a clear P300 response peaking around 400 ms with an amplitude of 24 μV. An unpaired *t*-test procedure confirms that target P300 amplitudes are statistically different from nontarget P300 amplitudes as this test rejects the null hypothesis at a 5% significance level (*p* = 0.0285).

## 5. Discussion

The previous results supported the idea that the conductive silicone material seems to be suitable for EEG recordings. Indeed, despite the significant difference in terms of impedance, ASSR SNR results obtained with gold-plated electrodes and conductive silicone electrodes are comparable. Additionally, the fact that the target ERPs evoked a large P300 supports the idea that the EARtrodes seem to be a promising candidate for future small, mobile, and unobtrusive BCI platforms. Furthermore, ASSR SNR results validate the capability of CochlEEG in recording EEG data with a sampling frequency of up to 4000 Hz. Future work should address the following outstanding issues in particular:

- The definition of the discomfort induced by in-ear devices has been the subject of debate for many years in the literature [41,42,43] and the comfort evaluations presented in this paper are not intended to tackle this question as several parameters (e.g., short wearing time and lack of activities performed by the participant while evaluating the comfort, among other considerations) narrow the conclusions that could be drawn from this limited study. However, even if a more extensive study is needed to conclude about the potential discomfort induced by wearing such in-ear devices, the comfort evaluations performed in this paper seem to indicate that there is no real difference between the custom and the generic in-ear piece, in terms of comfort. Nevertheless, several modifications should be done to improve the in-ear piece design. Indeed, according to the method proposed in this paper, there is more than one optimal position on the intra-aural earpiece where the electrodes could be placed; however, only one was tested in this study for each participant. Other positions should be tested individually or together in order to find the best possible electrode configuration. Furthermore, a handle to pull the earpiece out of the ear canal and a sound bore to let pass the auditory stimulation should be considered for mold design improvements.
- Even with the second behind-the-ear design, some difficulties remain the same for a few participants as to them not achieving acceptable electrical impedance at the point of skin–electrode contact. Therefore, the geometry and shape of the behind-the-ear piece should be further improved by increasing the flexibility of its structure in order to solve the problem of poor electrode-skin contact observed for a few electrodes.
- Results obtained with conductive silicone electrodes were compared with those obtained with gold-plated electrodes. Silver-plated (Ag/AgCl) electrodes are a good alternative to gold-plated electrodes. Such electrodes seem to be slightly more stable electrically, but need replacing more frequently because the plating layer (AgCl on top of Ag) is thin. Future work should investigate how silicone conductive electrodes perform compared to silver-plated electrodes.
- The impedance measurements were achieved using a medical grade impedance meter (Grass Technologies Electrode Impedance Meter), which does not provide measurements over a wider frequency range. Consequently, the electrode characterization presented in this paper remains limited. A thorough investigation over a wider frequency range should be conducted to complete the electrode characterization and to find out how the electrode-skin impedance might vary with time.
- While the EARtrodes have been validated with wet electrodes made of conductive silicone, the use of dry conductive silicon electrodes should also be investigated. Indeed, such dry electrodes would not require the abrasion of the Stratum Corneum and the use of electrolytic gel to reduce the skin’s impedance, which would make them easier to use and more acceptable in social settings [44,45,46]. Dry EARtrodes would also be great alternatives to semidry electrodes, which are not really user-friendly due to their cumbersome design [47,48,49].
- The major concerns for most composite materials tend to relate to the loss of durability and reliability over time. Therefore, a more comprehensive study should be conducted to evaluate the conductive silicone material’s aging profile, including physical and thermal performance over time.

## 6. Conclusions

The EARtrodes were presented in this paper as a lightweight and inconspicuous ear-centered device with the intention of enabling future innovative mobile EEG applications. The performance of the minielectrodes made of conductive silicone embedded in the device was evaluated with a custom 8-channel portable EEG research platform under two electrophysiological paradigms (ASSRs and ERPs). The ASSR results prove the capacity of the minielectrodes made of conductive silicone to reliably record good quality EEG data. The ERP results demonstrate that the ear-centered device can record a reliable P300 response and differentiate between target and nontarget stimuli responses, making it suitable for data acquisition for various applications in BCI like P300 spellers, among others. A more extensive ERP study, with a higher number of participants should be conducted to improve our generalization capability and better adapt to intersubject variability, an important parameter to consider in future BCI applications and machine learning algorithms.

In-ear sensors seemed to be relatively well tolerated by the participant whether they were embedded in a generic or custom-fitted earplug. Consequently, a product based on such technology would be a highly innovative earpiece able to noninvasively monitor diverse biosignals. Such BCIs have the potential to become the ultimate wearable technology with applications in the fields of neurogaming, military and telemedicine. Many research activities aiming towards a more comprehensive understanding of human brain function under natural conditions may also benefit from such technology, thanks to applications in the area of assistive technologies, neurorehabilitation and ubiquitous healthcare.

ASSRs results obtained at 4 kHz prove the capacity of CochlEEG to reliably record EEG signals with a 4 kHz sampling frequency. Consequently, when merged with CochlEEG, EARtrodes also benefit from high sampling rates, which opens up new possibilities for brainstem applications such as the automatic adjustment of hearing aids based on speech-evoked Frequency Following Responses (FFR) [50] or the fast diagnostic of brain-traumatic injuries based on FFR classification that would help to overcome the limitations of current diagnostic tools, which are time-consuming and require access to a nearby clinical facility and trained medical specialists [51].

## Figures and Tables

**Table 1:** Number of exploring electrodes used for each participant.

	S1	S2	S3	S4	S5	S6	S7	S8	S9
Number of exploring electrodes	3	3	3	3	3	1	3	2	2

## Data Availability

Data sharing not applicable.

## Abbreviations

The following abbreviations are used in this manuscript:

ASSR	Auditory steady-state response
BCI	Brain–computer interface
dB	Decibel
EEG	Electroencephalography
ERP	Event-related potentials
FFT	Fast Fourier transform
HL	Hearing level
HRA	Rockwell A-scale hardness
ICA	Independent component analysis
MMN	Mismatch negativity
RMS	Root mean square
TMJ	Temporomandibular joint
